# Cost Modelling for Recycling Fiber-Reinforced Composites: State-of-the-Art and Future Research

**DOI:** 10.3390/polym15010150

**Published:** 2022-12-29

**Authors:** Essam Shehab, Arshyn Meiirbekov, Akniyet Amantayeva, Serik Tokbolat

**Affiliations:** 1 Mechanical & Aerospace Engineering Department, School of Engineering and Digital Sciences, Nazarbayev University, Astana 010000, Kazakhstan; 2 School of Architecture, Design and the Built Environment, Nottingham Trent University, Nottingham NG1 4FQ, UK; 3 Civil Engineering Department, Faculty of Engineering, University of Nottingham, Nottingham NG7 2RD, UK

**Keywords:** cost modelling, cost estimation, composites, carbon fiber, recycling

## Abstract

Fiber-reinforced composites, such as carbon and glass fibers, are widely used across various industries. This is mainly a result of their outperforming properties in contrast with traditional materials. As a response to the environmental legal enforcement of the recycling of composite materials, several recycling methods such as mechanical, thermal, and chemical recycling, have been developed. Despite various merits, these recycling methods still face challenges, such as the heterogeneity of material, the quality of the recycled product, the high cost of recycling, and a lack of an established market. Since, in many cases, the financial aspect tends to be the major barrier to recycling composites, the appropriate cost modelling of the recycling process requires urgent consideration. To the knowledge of the authors, there is no prior research efforts on the reviewing of cost modelling techniques on composites recycling. Cost modelling research projects for different recycling technologies, with their findings and limitations, are sought from the literature and reported in this paper. It is found that recycling techniques still cannot compete with traditional landfilling in terms of cost, and are dependent on fiber recovery rates and plant capacities. Following a comprehensive literature review, research gaps are identified to formulate the research directions in this field.

## 1. Introduction

Composite materials have become widely used in various industries due to their superior properties, such as higher strength, lower weight, and resistance to corrosion, etc. In particular, composite materials are extensively used in the transportation sector due to their lower level of energy consumption and respective carbon emissions. For instance, composites currently comprise the largest share of materials (by weight) used in a new generation of commercial aircrafts, such as the Boeing 747 Dreamliner (50%) and the Airbus A350 (53%) [1,2,3]. The application of composites is also taking place in the automotive industry due to their weight-saving, which lead to improved fuel efficiency. Overall, composites offer an attractive alternative for industries where steel and aluminum are widely used. The strength of composite materials is reported to be higher compared to steel and aluminum but still, they weigh much less, resulting in 25 to 70% weight reduction. These qualities of composites expanded their application across different industries, such as transportation, automotive, aerospace, wind energy, and construction, etc. [4]. The composites market is dominated by glass fiber, with the global demand of 5.3 billion metric tons in 2019, two-thirds of which is consumed by transportation, construction, and pipeline industries. In the same year, the global carbon fiber demand reached 100,000 metric tons, which was mainly driven by aerospace and wind turbine production industries [5]. The current state of the market distribution for composite materials is represented in [6].

The global market size for composites was reported to be almost 95 billion USD in 2021, with projection to reach nearly 126 billion USD by 2026 [7]. Researchers all around the world, along with industry members, are working on finding ways to minimize future and current waste that comes along this increasing use of composite materials.

Despite all the advantages, owing to their strong and durable characteristics, composite materials are hardly recyclable. The increasing rate of production of composite materials across industries led to the generation of substantial amounts of composite waste, both from production waste and end of life waste. For example, in 2016, in the UK, 30–50% of Carbon Fiber Reinforced Polymer (CFRP) or 2000–3000 tons/year was thrown away as waste [8]. As reported by the European Technology and Innovation Platform (ETIP), almost 700,000 tones of composite waste will be accumulated in next five years, mostly from the construction industry [9]. Boeing and Airbus production result in carbon fiber waste in the form of cured and uncured prepregs from the production of Boeing 787 and A350 XWB each year [10]. Overall, it is estimated that 12,000 commercial planes will be decommissioned by 2040 [11]. Each aircraft contributes 20 tons of carbon fiber-reinforced composite, while the transportation industry results in 214,000 tons of composite waste from discarded car waste [12]. This waste can no longer be easily and cheaply disposed in landfills due to established strict tax policies and legislation. For example, landfilling composites can cost from 5 EUR/ton to over 100 EUR/ton [13]. Additionally, 85% of all the weight of any decommissioned end-of-life vehicles (ELVs) has to be recycled, while 10% of it has to be recovered in terms of energy, by 2015 [14]. Another challenge in the composite market is that industry members are following stricter procedures for material selection, which implies using life cycle assessment, and questions the current composite waste management system.

Even though conventional materials such as metals, glass, and plastics, reached a high-level of recycling, the recycling of composite materials faces various issues with respect to their sustainable usage in multiple applications. According to [15], technical, supply chain, and immature market factors that restrict or delay the rates of recycling composite materials, and affect the willingness of manufacturers to engage, can be categorized as shown in Table 1.

The increased consumption of composite materials, and recent legislation tightening across various spheres, force the industries that use composite materials to consider various optimal routes for their recycling. This paper focuses on two key research areas, namely, “cost modelling” and “recycling fiber-reinforced composites”, and reviews the recent works that attempted to develop the cost models for composites recycling processes.

This paper is organized as follows. The process of literature review adopted in this study is explained in Section 2. Section 3 gives a brief introduction on the types of composite material depending on the reinforcement and matrix type. The most common and commercially available composite recycling techniques are introduced in Section 4. Section 5 explains different terms and methods used in the literature to assess the Life-Cycle Cost (LCC) of composites. Recent research attempts and their findings on the cost modelling of Carbon Fiber-Reinforced Polymers (CFRPs) are presented in Section 6. Finally, the paper is concluded by providing research limitations and future research directions.

## 2. Methodology

This section describes the methodology that was used to screen the relevant literature that studied cost and financial feasibility of recycling Carbon Fiber-Reinforced Polymer (CFRP) materials. This study aims to gather information about how cost estimation is carried out in the CFRP recycling industry, as it is a relatively new field that has been assessed sparsely previously. Cost estimation itself is very dependent on different variables and cannot be generalized across countries or time. Consequently, it is better if several studies are present to each recycling stage and method to be able to cross-validate results. In this particular study, the review on cost estimation models were gathered from two research areas i.e., “recycling cost modelling” and “recycling composites cost”.

**Figure 1:**
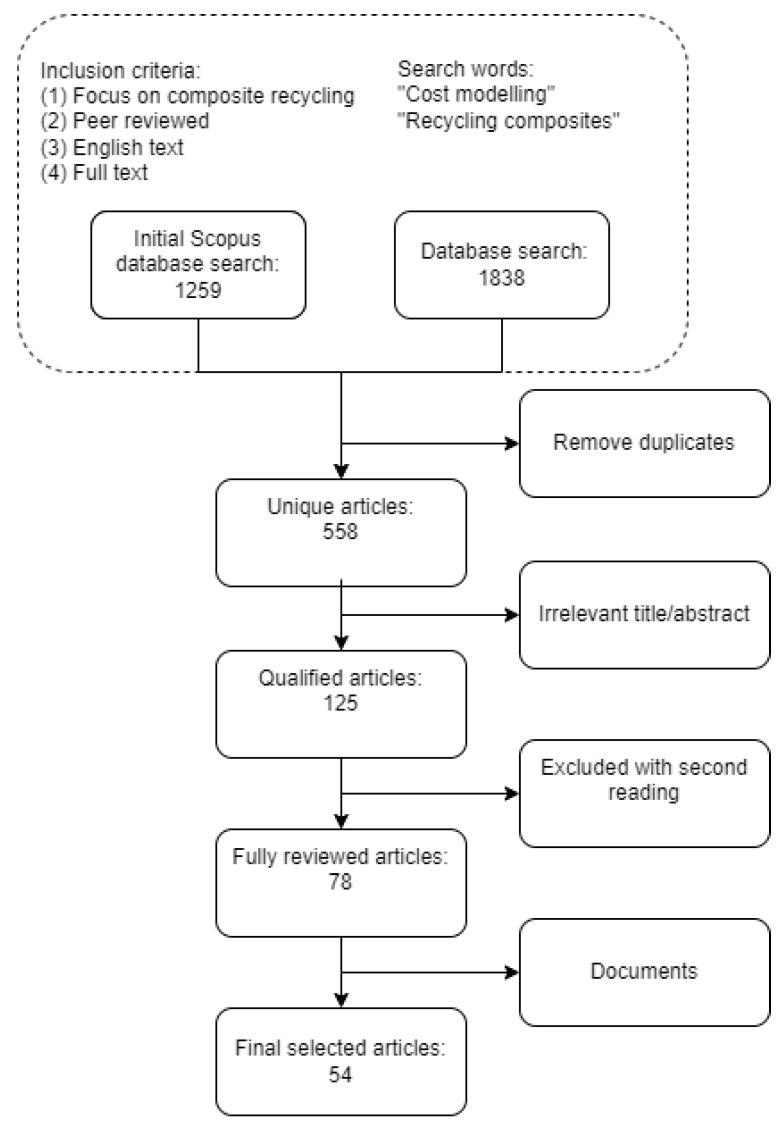
The literature review process.

The results of cost estimation studies cannot easily be compared to each other since system boundaries, data type, and data sources, are different in each case. However, the available studies allow to see the overall picture and patterns and might be used for further assessments. The methodology adopted for this research study is a traditional narrative-based review of relevant literature in the field described by Mayer (2009) [16]. The main aim of this work is to review the cost modelling related works in terms of recycling composites as opposed to reviewing technical aspects of those techniques. The main database used for the search of literature was Scopus, which was supplemented by other databases such as the Web of Science, ScienceDirect, Research Gate, IEEE Xplore and Google Scholar. Articles published only in English were included in this review. The keywords for the search of relevant studies were “cost modelling”, “cost estimation”, “financial analysis”, combined with (using a Boolean operator “AND”) the recycling related part of the research with keywords such as “recycling composites”, “recycling fiber-reinforced composites”, “recovery of carbon fibers”, “recycling glass fibers”. As a result, the relevant research articles were chosen based on the analysis of the title, abstract, and results of the study. Figure 1 shows the steps of the literature review with the number of selected and excluded articles. The main part of the work, state-of-the-art, does not include research works which did not address both research areas, namely, cost modelling and recycling composites. However, a few studies with no direct relationship to composites were included which would potentially provide further guidance for the research. The review includes only recent studies published during the last 20 years, as the maturity of the recycling methods was not at an adequate level prior to this period. The number of studies that ties the concepts of cost estimation and recycling composites was found to be limited.

## 3. Classification of Composite Materials

A composite material is a material produced by a combination combining two or more materials with significantly different physical properties, which allows obtaining desired unique properties. The combination of different materials allows to obtain new materials that are stronger, lighter, or less expensive, compared to conventional materials [17]. The composite material is generally a combination of the matrix and the reinforcement. According to the matrix type, the matrix materials are classified into metals, plastics, and ceramics [17]. Composite materials, in turn, can be classified by the types of reinforcement such as, for example, fiber-reinforced composites, particulate, and structural composites. The fibers can be classified according to their lay-up and length, such as continuous (aligned), long, or short [18].

**Figure 2:**
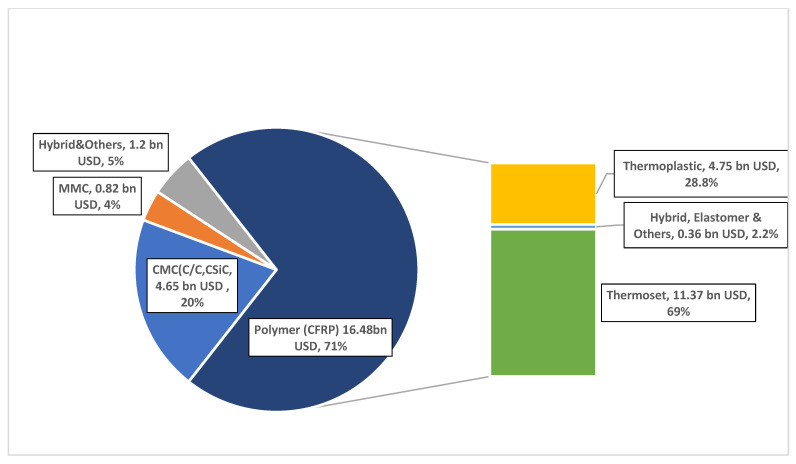
**Key:** MMC = metal matrix composites; CMC = ceramic matrix composites; CFRP = carbon fiber-reinforced polymer. Carbon composite’s market share segmented by matrix material (reproduced from [22]).

Based on the polymer type, fiber-reinforced composites are divided into thermosets and thermoplastics [19]. The fundamental difference between thermosets and thermoplastics is that the first type of material is cross-linked and cannot be remelted, while thermoplastics can be melted and reshaped [20]. Polymer-matrix composites cover the vast majority of the composites market, in which two-thirds correspond to thermoset composites, though the application of thermoplastics composites has increased significantly in recent years [21]. Another recent report by Sauer and Kühnel (2018) [22] indicates that thermosets still correspond to 69% of all carbon fiber composites, as illustrated in Figure 2.

**Figure 3:**
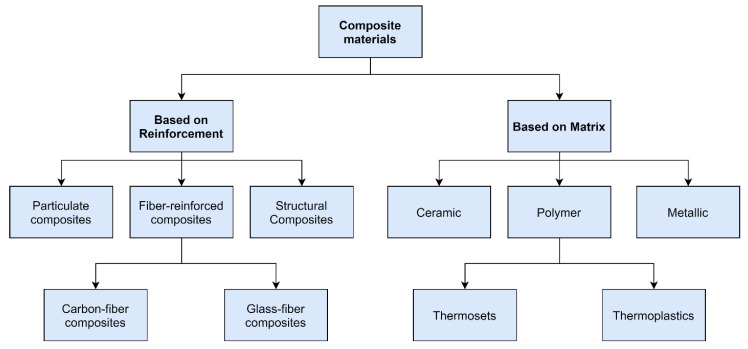
Composites classification based on the reinforcement and the matrix type.

Thermosets have been used widely due to their superior mechanical properties, higher-temperature resistance, affordable prices for the manufacturers, and diverse matrix systems and producers compared to other alternatives. Nevertheless, the thermoplastics have their advantages in terms of short processing time, damage tolerance, and recyclability. It is projected that these properties will enhance the attractiveness of thermoplastic composites in the market [22]. The overall classification of composite materials based on the matrix and the reinforcement type is shown in Figure 3.

The scope of this paper was assumed to be limited to the carbon and glass fiber-reinforced composites with polymer matrix, due to their predominance on the market. 

## 4. Review of Recycling Technologies for Composites

**Figure 4:**
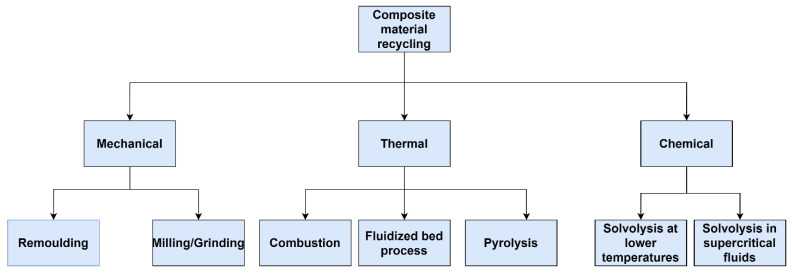
Classification of composite material recycling methods.

Pakdel (2021) [23] provided a comprehensive review of a recent advancement of CFRP recycling methods. A broad review of the recycling techniques with the focus on further application of recycled materials was conducted by Gopalraj and Kärki (2020) [24]. An extensive review of the most recent research works on recycling carbon fiber composites was made by Beatrice Colombo (2022) [25]. Most of the recycling technologies, according to the studies above, were mainly focused on polymer-matrix composites due to their large share on the market. Additionally, due to technical difficulties associated with the separation of thermoset matrix such as epoxy resin, a great share of the literature was concentrated on the methods of recycling thermoset composites. These and other challenges and limitations associated with CFRP recycling are described by Meiirbekov et al. (2021) [26]. The recycling technologies used for thermoplastic and thermoset composite materials are shown in Figure 4. Mechanical, thermal, and chemical recycling, were identified as proven methods for thermoplastic and thermoset composites and were described in detail in the following sections.

### 4.1. Mechanical Recycling

Remolding or remelting are used only for thermoplastic matrix composites because of their important characteristic of being able to be reshaped under high temperatures. The main challenge related to the thermoplastic composites is their viscosity at the melted state (500–1000 Pa·s), which is considerably higher than for thermosets (less than 100 Pa·s) [27]. The process requires high-pressure tooling for the impregnation of fibers and energy inputs for temperature control of this tooling, which makes the process rather expensive. Recycling methods for thermoplastic materials are studied more in terms of non-composite polymers rather than in the form of composites. The following methods will not include thermoplastic composites and focus on only thermosetting polymers [21].

Mechanical recycling is a conventional method of recycling thermoset matrix composites, in which the material is reduced in size by the process of grinding, cutting, milling or shredding operations [28]. Initially, the material is cut into small pieces of 50–100 mm size and fed into shredder after manual removal of inserts. Further, these pieces are grounded into particles with the size of 10 mm to 50 μm. The larger particles are used as a filler material in bulk mounding compounds (BMCs), while the smaller particles are utilized in sheet molding compounds (SMCs). Several companies realized this method successfully, such as, for example, ERCOM in Germany and Phoenix in Canada [20]. Recent studies by Pietroluongo et al. (2019) [29] analyzed the effects of mechanical recycling on glass fiber composites in the automotive industry. The study found that even though the mechanical properties of materials were degraded during the recycling process, the secondary product can be still be used in less critical automotive applications.

Mechanical recycling offers a few advantages, such as, for example, the lack of using any hazardous materials during the recycling process and recovery of both fibers and resin [20]. The mechanical recycling of carbon fibers (CF) requires hundred times less energy in comparison with manufacturing virgin CF [24]. However, various limitations made the prevalence of this method impossible in the market. This is because recycled products can only be used as powders or short fibers, which limits the area of application of the product. Their mechanical properties are degraded and can be used only as fillers in new composites [30]. The competitive advantage of such a recycled product is questionable with the availability of cheap calcium carbonate, which is the main filler in SMCs and BMCs [20]. Finally, the processes involved in the recycling process are energy-intensive, which significantly increases recycling costs [31].

### 4.2. Thermal Recycling

The incineration of used composites or combustion for energy recovery cannot be considered as a recycling process since these processes do not result in the recovery of the material itself. Therefore, there are only two thermal methods of recycling composites, i.e., (a) fluidized-bed recycling process and (b) pyrolysis. Both methods have been studied extensively and was found to have great potential on the market. Each recycling method has its own advantages and disadvantages; therefore, the choice of the recycling method depends on the fiber type, the matrix, and the desired properties of the final product. Thermal recycling is mostly chosen for unknown or mixed materials [20].

#### 4.2.1. Fluidized-Bed Process

A fluidized bed thermal process was developed at the University of Nottingham, UK, to recover glass and carbon fiber composites. During the process, the material is prepped by reducing its size to 25 mm before feeding it into a fluidized bed. The bed consists of silica sand that is fluidized by applying an airstream at a temperature range of 450–550 °C and velocity of 0.5–1 m/s. As a result, the polymer matrix is vaporized allowing to release fibers and fillers out of the bed as individual particles via gas flows. The individual fibers are then separated from the gas stream using a cyclone, after which the residual polymer is oxidized in a secondary chamber. The energy generated during the combustion process can be reused [20]. This method is optimal for highly contaminated composites with metal inserts as heavy components sink during the process [30]. The study by Pickering et al. (2015) [32], which examined the fiber recovery by the fluidized bed process in the laboratory scale, found that up to 18% of the tensile strength of the material is lost after the recycling process, although the stiffness remained the same at 70 GPa. This result was deemed to be promising as the critical constituent for potential applications of recycled short fibers is stiffness [33]. In a similar study, the authors were able to preserve 75% of the tensile strength of fibers and clean surfaces comparable with virgin fibers during the recovery of carbon fiber composites using a fluidized-bed method [34].

To conclude, the fluidized bed thermal process provides several advantages compared to its alternatives, such as, for example, tolerance for contaminated materials and the recovery of energy. In addition, the surface of fibers remains to be clean and does not result in oxidation allowing a bonding in a polymer matrix for reusing as a composite [20]. With respect to limitations, the fluidized bed thermal process results in short fibers with degraded mechanical properties of the product, which limits its potential areas of application.

#### 4.2.2. Pyrolysis

The pyrolysis technique is widely applied in the recycling of composites, particularly for the recovery of carbon fiber composites [35]. Pyrolysis is the thermal processing of organic materials in the absence of oxygen. During pyrolysis, the composite material is exposed to high temperatures in the range of 450–750 Celsius forcing the matrix to decompose to lower-weight molecules. At the same time, fibers are not affected and can be recovered [30]. This matrix decomposition leads to the creation of oil, gases, and solid particles (fillers and char). The resulting gases during the process mostly consist of hydrogen, methane, and other hydrocarbons, which can be used for energy recovery.

According to Morin et al. (2012) [31], the degradation of mechanical properties for pyrolysis ranges between 4–20% in contrast to virgin carbon fiber properties. The degradation rate of fiber composites’ properties is highly dependent on the parameters used during the process. For example, mechanical properties of glass fibers were significantly affected, losing at least 50% of mechanical strength even when processed at 450 °C [4]. A recent study on glass fiber composites showed that it is possible to reduce the negative impact of the temperature by using a two-step temperature pyrolysis process up to 19% of the tensile strength and 43% of the flexural strength of the glass fiber, compared to single-step pyrolysis [36]. Carbon fibers are less affected by high temperatures. However, if the temperature and oxygen are not controlled, the surface of fibers can be covered by pyrolytic char during decomposition requiring additional processing [4]. Several studies attempted to eliminate the residual char and recover original mechanical properties. The methods they adopted include variations of the pyrolysis process using a catalyst, vacuum, and microwave pyrolysis processes [31]. Among these methods, only microwave and catalytic pyrolysis processes resulted in both preserving mechanical characteristics and clean surfaces of carbon fibers [31].

In the process of microwave pyrolysis, the traditional heating system is replaced by the microwave radiation. According to Obunai et al. (2015) [37], 100% of resin was eliminated using 700 W argon atmosphere and recycled carbon fibers lost only 0.7% of tensile strength. Zhang et al. (2020) [38] and Naqvi et al. (2018) [39] carried out a critical review of pyrolysis conditions, challenges, and its reuse applications. The process was reported to be the most practical and sustainable recycling process prior commercialization.

In general, the pyrolysis technique provides a wide range of advantages. First of all, it does not require applying chemicals, and energy from the gases can be used to heat the chamber directly [20]. Furthermore, this method is energy efficient and requires only 5–10% of the energy compared to virgin carbon fiber. Although the process parameters can affect the resultant characteristics of fibers, they can still be recovered to the levels close to virgin fibers. According to Guo et al. (2019) [40] recycled carbon fiber composites with epoxy resin were able to demonstrate properties close to virgin fibers. The potential area of recycled products application is short carbon fibers in brake pads. With respect to glass fiber composites, their strength degradation does not allow using them in structural applications, however, they can be suitable as thermal insulation materials [4].

### 4.3. Chemical Recycling

The chemical process of recycling composites is called solvolysis. In the solvolysis technique, the polymer matrix is degraded by exposure to a solvent. Solvolysis can be categorized as (a) solvolysis at lower temperatures, and (b) solvolysis in supercritical fluids depending on the temperature and state of the solvent. There are also other classifications of solvolysis based on the acting solvents such as, for example, hydrolysis (water), glycolysis (glycols), and acid digestion (using acid).

Solvolysis at lower temperatures is the process of chemical decomposition of a polymer matrix by means of the application of reactive substances such as alcohol or glycol. The result of the process is the fibers, inorganic parts, and remaining solvent. Liu et al. (2004) [41] conducted a study in which the carbon fiber composite with epoxy resin matrix has been dissolved in nitric acid. The fibers maintained the mechanical properties and lost only 1.1% of the tensile strength. Solvolysis has been proven as one of the most efficient recycling methods in terms of preserving properties of fibers amongst others. Another distinctive feature of solvolysis lies in the fact that it can regenerate both fibers and depolymerized matrix into monomers or petrochemical raw material [21]. However, the solvents applied during the process can cause a negative impact on health and the environment. Moreover, the solvolysis technique is sensitive to contaminants such as, for example, metal inserts, which need to be removed before the recycling process. According to Oliveux, Dandy, and Leeke (2015) [4], numerous lab-scale experiments were carried out to study the solvolysis at lower temperatures, but only a few were able to progress to the industry-scale, thus, creating demand from the industries. For instance, Panasonic Electric Works was willing to recycle 200 tons of glass fiber composites using hydrolysis [42].

The solvolysis of composites in supercritical fluids is another popular recycling technique, which was developed in Japan in 1995. The supercritical fluid has a temperature and pressure exceeding its critical point with an intermediate phase between liquid and gas. They tend to have low viscosity, high diffusivity, and pressure-dependent solvent power. A study by Okajima et al. (2002) [43] were the first to use supercritical water at temperature range of 300–450 °C to process carbon fiber-reinforced composite with the epoxy resin matrix. The recycled fibers resulted with 10% lower tensile strength compared to virgin fiber. Another study by Jiang et al. (2009) [44], in turn, tested the supercritical alcohol at 310 °C to recycle carbon fiber-reinforced composites. The recovered fibers have preserved mechanical properties and clean surface at the levels comparable to virgin fibers.

A recent study by Henry et al. (2016) [45] conducted an experiment by mixing water and methanol for recycling carbon fiber-composite with epoxy resin. The use of a mixed solvent allowed to achieve better results compared to pure water and created recovered fibers with properties comparable to virgin CFs. In general, the application of supercritical fluids proved to be a promising solution in terms of recycling composites with polymer matrix. However, parameters of the process such as temperature, pressure, and time, must be controlled with due consideration of the composite types. Another disadvantage of applying supercritical fluids is that solvents at supercritical conditions require mode advanced equipment to endure high temperatures, pressures, and corrosion, all of which makes the recycling process more expensive in contrast to solvolysis at normal conditions [46].

The recycling techniques described have their own advantages and disadvantages, but they conceal a wide range of associated costs. Various factors such as the achievable recovery rates of fibers, the scalability of the recycling process, capital costs of equipment, etc. might affect the final decision by industry players on the recycling technique to be adopted.

In recent years, new technologies such as AI and IoT are proposed to be applied in the CFRP recycling industry to make it more advanced and cost-effective. Some of the ideas of such application are provided in the study by [47].

Section 5 covers the details and definitions of these cost factors to provide a better understanding of the cost modelling frameworks in composites recycling. Apart from that, the sustainability concerns of composites recycling are discussed in the next subsection.

### 4.4. Sustainability Considerations

As mentioned previously, at the global level the processes of production and consumption of composite materials is continuously increasing. Although the use of such materials has been known in the past, their more recent application is gaining momentum both due to the demand from various industries and the notable cost decrease over the last few decades [48]. Several types of composites have been inflowing the various industries and replacing traditional materials from the market. This trend is reported to be continued across all of the categories of composites [49].

Despite all the advantages that these materials provide, there is a growing concern regarding the waste generated from their application. For instance, the waste can come from rejected new products that use composites or from the end-of-life products. Since the latter is concerned with the 20–25 life years’ span, the present-day waste generation pressure becomes more prominent than in previous years. The waste output in terms of all types of composites is increasing and projected to grow both across industries and across borders [50,51]. The recycling of composite waste, and especially carbon fibers, is a way forward in addressing the posed issue. The recycling techniques presented and discussed in this study clearly support the idea that recycling is the way to economic and environmental sustainability. However, pure reliance on the fact that recycling is sustainable can be insufficient.

In order to clearly understand the true adherence of the recycling methods to sustainable development principles, it is important to look at various indicators, including socio-economic and environmental indicators, and many more sub-indicators under these three main pillars of sustainability. However, sustainability assessment can be confusing to the decision-maker due to the availability of a wide array of sustainability indicators [52]. A study by Pillain et al. (2017) [52] has attempted to analyze the existing indicators and combine them for the application for the carbon fiber recycling sector. The conducted literature review and following analysis helped to identify the most relevant indicators. For the environmental category, indicators such as “global warming”, “acidification”, and “human toxicity” were prioritized, whereas for the socio-economic category indicators such as “supply risks resulting from resource deficit” as well as “supply disruption due to geopolitical issues” were identified as the most relevant. These indicators are of a higher level and should be looked at from a broader perspective. Another study, by Ribeiro et al. (2016) [53], investigated the indicators of a specific nature that could practically improve the recycling process by optimization or improvement at a case study project. They have identified indicators such as savings on electric energy, reduction on warm-up periods, and replacement of the die heating system. The latest study by Meiirbekov et al. (2022) [54] identified the challenges and provided recommendations on the environmental assessment of recycling techniques. The authors also listed possible environmental indicators for the Life Cycle Inventory (LCI) analysis of the CFRP material. Further research should investigate the sustainability aspects more closely, especially when the cost modelling is concerned.

## 5. Life-Cycle Assessment of Composites Recycling

**Figure 5:**
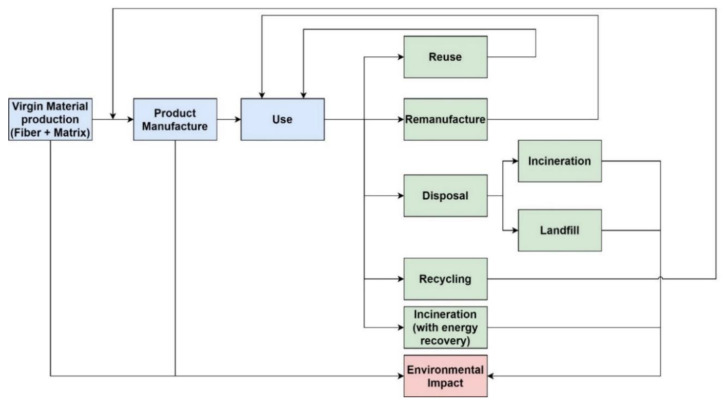
A life cycle of composite materials and recycling routes.

Many studies identified in the literature which estimate the costs of recycling composites, refer to the LCC analysis. The LCC process considers all the costs starting from the design stage and ending with the disposal phase of a product. The recycling of composites requires more in-depth consideration of the process costs and comparative analysis of different disposal routes. For this purpose, Figure 5 illustrates the product life cycle and recycling routes for composite materials.

The production of virgin fiber composites, products manufacturing including composites and disposal, requires the conversion of material and energy resources. These operations cause a negative environmental impact including the issues of energy consumption, air pollution, and waste management. The Life Cycle Assessment (LCA) framework has been developed to evaluate the energy consumption and environmental footprint of a product or a process. The methodology is validated and standardized by the International Organization for Standardization (ISO) in ISO 14040 and 14044 standards [55]. LCA focuses on the entire life cycle of a product or a process, starting from the raw materials and ending with the disposal stage. Numerous studies adopting the LCA approach were conducted to evaluate the recycling of composites in terms of energy and environmental impact and predicted a promising future throughout their life-cycle both during application and recycling [55,56,57,58].

Figure 5 (green colored boxes) indicates that the end-of-life products containing composite materials have the following potential routes:

Reuse—reusing the composite material may not be practical for some applications. For this route, manufacturing processes must be adapted in a way that the design will allow the material re-use [59];

Remanufacture—some products from composites can go through restoration processes, which allow regaining their original properties and performance;

Disposal—this process refers to the elimination of waste without extracting any value from it;

Recycling—recycling is acquiring a value from the end-of-life products at a material level [60]. In contrast to remanufacturing or reuse, in the case of recycling the form of the product is reproduced, and materials undergo reprocessing. Incineration with energy recovery is not considered in this category as it does not result in the recovery of a material.

The aforementioned pathways for fiber-reinforced composites are critical to consider, as different studies that were reviewed in this article focused on different recycling or disposal pathways including supply chain [60]. For instance, the study by Farel et al. (2013) [61] have developed a model which can be used to evaluate the feasibility of glazing recycling. Whereas the research work of Witik et al. (2011) [62] limited on the LCA approach to the energy recovery from incineration. The reviewed literature found that the waste treatment steps in the lifecycle of fiber-reinforced composites were poorly studied. While the majority of works adopting LCA emphasize the production phase rather than utilization [62,63,64,65], the literature review also revealed that the previous studies referred to different stages of composite products. For example, Meng, et al. (2018) [66] provided the cost model, which considered three phases namely: (a) the fluidized bed recycling phase; (b) the processing and manufacturing of recycled carbon fiber (rCF) automotive components; and (c) the application phase, in which weight savings of automotive components are taken into account. On the other hand, another study by Li, et al. (2016) [67], which compared the financial viability of the mechanical recycling process with landfilling and incineration, included only the recycling phase. The authors decided to focus only on assessing recycling and disposal routes excluding costs incurred during production and application phases. The scope of this paper is limited to studies which examined the recycling phase cost models, and which may contain information on costs for other phases.

Different cost categories that can be incurred during the recycling of composite materials have been explained for clarity purposes. The costs incurred during recycling can be categorized as: (1) capital expenditure (CAPEX) and operational expenditure (OPEX); (2) variable and fixed costs; (3) direct and indirect costs [68].

The CAPEX is generally incurred during the acquisition of fixed assets. It is the initial volume of investments necessary to start a business. These expenses are usually documented as an asset rather than an expense, which can depreciate over time [69]. In terms of recycling processes, this might include the costs of equipment and tooling, and manufacturing building costs. Equipment costs may significantly vary from process to process. OPEX are typically incurred in the process of regular recycling activities. These costs are part of day-to-day operations, such as dealing with raw materials, utilities, maintenance, labor, plant overheads, maintenance, etc. Additionally, there might be indirect costs such as insurance, administrative costs, and depreciation [68].

A direct cost is an expense that can be associated with a specific object, such as an activity, a product, or a project. Therefore, it is usually easy to identify direct costs and associate them with their end-result such as a product, a service, or a function. Some of the examples of direct costs for recycling activities can include direct labor, materials, and maintenance costs, etc. On the contrary, indirect costs cannot be directly linked to their end objectives, whether it is a product, a function, or a project [68]. Differentiating these costs is critical in examining the costs of recycling processes. The examples of indirect costs associated with recycling activities among others are insurances, administrative costs, quality control, and depreciation. Usually, direct costs are deemed as variable costs, while indirect costs tend to be perceived as fixed costs [70,71].

## 6. Recent Attempts of Cost Modelling for Recycling Fiber-Reinforced Composites

Recycling composites has a positive impact on the environment specifically in terms of reducing emissions and energy consumption. The financial viability of recycling processes depends on many parameters and case-specific factors that authors exploited in their studies. The list of studies reviewed in this work along with the methods involved and result descriptions are summarized in Table 2.

Table 2 represents the most recent works related to the costs of recycling carbon and glass fiber composites ordered chronologically. The authors scrutinized the different recycling methods and compared them with the traditional disposal routes such as landfilling and incineration. Some of the works covered several techniques and compared their costs with each other [58,72,74]. On the other hand, other works focused on a single recycling technique and a specific type of waste, namely automotive components [73,77]. Generally, the authors provided a sensitivity analysis in which the variation of certain features such as the final recyclate value, the output rate of production, fiber recovery rates are examined to assess their impact on the final costs. The majority of papers related to cost modelling of recycling composites mainly focused on carbon composites, and this could be explained by the high value of the product. Hedlund-åström (2005) [72] was one of the pioneers in a new century who attempted to conduct LCC and LCA for recovering CF waste. It was found that the disposal route for CF waste should be chosen based on the final application of rCF. Incineration was found to be attractive compared to recycling for cases when rCF is needed to replace low-value materials such as glass fiber. The authors provided energy consumption rates for the mechanical processes of recycling, which was further used in other studies [72].

**Figure 6:**
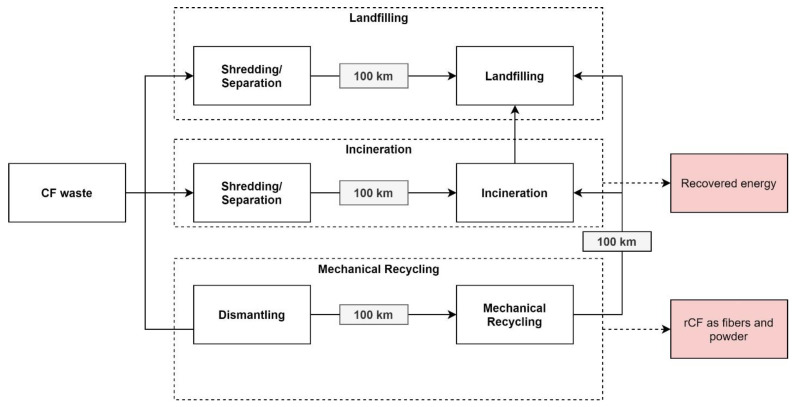
Waste treatment routes analyzed by [73], with permission from Elsevier, 2022.

Li et al. (2016) [73] developed both LCA and LCC models to assess the environmental and financial feasibility of the mechanical recycling process for carbon fiber composites. Additionally, incineration and landfilling pathways were included in the analysis to examine environmental and financial effects. The LCA included modules of different disposal options such as landfilling, incineration, and mechanical recycling. The used model representation is illustrated in Figure 6.

The study of Li et al. (2016) [73] did not include the costs of production and the use phases of CF components, as costs during these stages remain the same regardless of the chosen waste treatment route and can be excluded. The study also included the costs of dismantling components (1.38 GBP per kg), which is needed before starting the recycling process. Additionally, CF waste was pre-treated by shredding with other automotive parts. Transport distance of 100 km were considered for a more realistic scenario of transportation of the waste between dismantling or shredding and a recycling site. All costs related to landfilling and incineration were estimated considering pre-treatment costs, costs for transportation, and fees at facilities. A limitation of this study is the fact that the revenues from selling energy recovered in the incineration process were not included in the model. As far as the recycling process concerned, the capital costs were calculated based on a study by Hedlund-åström (2005) [72]. According to this study, recovery rates for fibers were low (40%) and the market values of rCFs were not sufficient at this point. Higher recovery rates of fiber composites and potential high-value applications (vCF displacement) might improve the financial viability of the recycling technique. In terms of other disposal routes, the authors suggest that landfilling tax has encouraged the shift of CF treatment from landfilling to incineration resulting in landfilling costs of 220 USD/ton in contrast to incineration cost of 190 USD/ton of CF waste.

Meng et al. (2018) [66] developed a model that aimed to assess the viability of recycled carbon fibers using a fluidized bed process in automotive applications. The model assessed life cycle costs in the following stages: (1) the recycling of CFs with the fluidized bed process; (2) the processing of CFs; (3) the manufacturing of components from rCF; and (4) use phase, during which cost savings are taken into account in terms of fuel consumption due to substitution with lightweight rCF.

For the recycling phase, capital and operational costs were assessed for a plant with a theoretical capacity of 1000 tons per year and the sensitivity analysis included the range of 100–6000 tons per year. Various manufacturing routes were investigated including a few fiber structures (random, aligned) and volume ratios for compression and injection molding processes. Processing costs for the wet papermaking process for randomly structured rCFs and fiber alignment process costs were included as energy and labor costs. The application phase was included to consider weight savings due to CFs usage compared to traditional materials. Overall, all life cycle costs were compared to conventional materials (steel and aluminum) to analyze the relative performance of rCFs. According to Meng et al. (2018) [66], CF can be recovered at USD 5 per kg, which corresponds to 15% of the cost of virgin CF. The performed sensitivity analysis presented that this cost can be reduced if key process parameters are upgraded such as plant capacity or feeding rate per unit bed area. Aligned rCF composites significant cost reductions in contrast to virgin CF and conventional materials, such as steel and aluminum, are primarily due to reduced fuel consumption. However, the cost model developed by the authors did not mention the sorting, dismantling, and transportation costs of CF material to the plant, which could affect the results of the study, and thus be considered as a limitation of the study. For instance, the cost of Boeing 747 airplane dismantling may vary from 60,000–120,000 GBP which is approximately 0.33–0.65 GBP per kg of CF waste [78].

**Figure 7:**
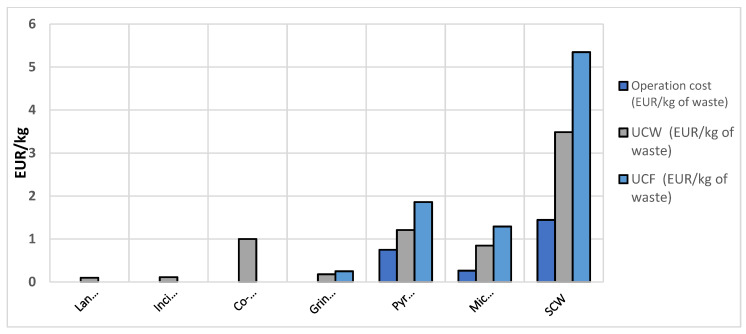
**Key:** UCW = unit cost per mass of unit waste; UCF = unit cost per mass of recovered fiber; SCW = super critical water. Economic indicators for assessed routes [58], with permission from Elsevier, 2022.

Vo Dong et al. (2018) [58] conducted an economic and environmental assessment of disposal routes for CF waste. The developed model compared landfilling, incineration, mechanical recycling, pyrolysis, and chemical recycling (supercritical water (SCW)) methods in terms three economic indicators, namely: (1) operation cost per mass of unit waste; (2) the unit cost per mass of unit waste (UCW); and (3) the unit cost per mass of recovered fiber (UCF), as shown in Figure 7. Vo Dong et al. (2018) [58] explained that co-incineration allows material recovery in addition to energy recovery. In co-incineration, waste is used as a substituted fuel involved in clinker fabrication where coal is normally used as a fuel and the products of waste combustion, i.e., heat and ash, are completely valorized in co-incineration. SCW is the recycling technique which focuses on the utilization of water, a cheap and low-hazardous risk raw material compared to organic solvents, but this technique requires a large amount of energy to operate at supercritical conditions [58].

Despite having some limitations, such as the lack of transportation, conditioning, and packaging costs, the authors provided a comprehensive assessment of all mentioned recycling and disposal routes. According to the study, recovery pathways still cannot compete economically with the traditional ways of disposal. Solvolysis in supercritical water was found to be the most expensive process, which was followed by pyrolysis and microwave techniques. The UCF indicator was used as a critical measure to compare with reported market prices for rCFs, virgin CFs, and glass fibers. All UCFs resulting from recycling techniques (except for solvolysis) were lower compared to data. Nevertheless, it must be emphasized that the costs of recovered fibers still do not seem attractive compared to the market prices of vCFs for general purposes from cheap materials such as lignin.

Overall, the recycled fibers using commercially available methods allow to achieve around 200 GPa of elasticity, which market price is between USD 0–22 per kg [24]. In [58], the authors identified the preferrable price range (average unit cost per mass UCF) for recycled carbon fiber to be economically viable compared to virgin carbon fiber. The prices in the market might vary depending on mechanical characteristics, for example, from USD 20 per kg for low modulus to USD 2000 per kg for ultra-high modulus [79]. In the study [80], the authors conducted a cost-benefit analysis of CFRP waste recycling methods and came to the conclusion that solvolysis brings more profit compared to other methods; however, the performance is highly dependent on chemical solutions used. The pyrolysis method is also capable of returning a high profit. Table 3 shows the average tensile strength retention of recycling methods, processing temperature range, and UCFs.

While the former two studies [58,66] were used to assess the environmental and economic aspects of recycling CF composites using LCA, a study by Hagnell and Åkermo (2019) [74] focused on developing the recyclate value model, which aimed to assess the potential of closed-loop usage of fiber-reinforced composites such as carbon and glass fibers for structural applications. The costs fully focused on the production phase using recycled composite materials using pyrolysis, mechanical recycling, and co-processing in cement kilns. The main focus of this work, among others, was the intention to link the material values with mechanical performance after each cycle of specific recycling methods. The proposed loop with the variation of the value of recycling fiber and mechanical performance after each cycle has demonstrated that 50% of cost reductions can be achieved by using recycled fibers.

It is important to consider the economic assessment of recycling composite materials within the end-of-life elements, such as vehicles and aircraft, including dismantling, sorting, and transportations costs. To the knowledge of the authors, there is no study available in the filed discussing the calculation of recycling costs of composites within the whole item (vehicle, aircraft). However, Farel et al. (2013) [61] have developed a model which can be used to evaluate the feasibility of glazing recycling for end-of-life vehicles (ELVs). Cost and benefit analysis (CBA) methodology was applied, in which the authors tried to simulate the impact of variations in different exogenous factors, such as final recyclate prices, landfilling costs, and logistics costs, etc. Moreover, the study debated the challenges of glazing recycling at a national level and proposed potential solutions with evidence. The CBA of recycling glazing was based on the general recycling scheme of ELV materials, which made the study convenient to reproduce the results on other non-metallic materials including fiber-reinforced composites. Regarding end-of-life aircrafts, Zhao et al. (2020) [57] have developed an economic indicator that can help end-of-life recyclers with making a decision on the type of disposal scenario to adopt. The developed indicator is the ratio of the difference of salvage value (after the recycling process) and the residual value of an aircraft over the costs incurred for dismantling and recycling processes. The analysis of diverse strategies with real-life cost breakdown structures identified that the salvage value of an aircraft is an important aspect, which resulted in the negative economic indicators for dismantling and recycling processes of an aircraft’s bodies, whereas engine dismantling and recycling processes represented positive sums. It should be recalled that ELV and aircrafts have residual values, which should be included in the analysis.

## 7. Conclusions and Implication for Future Research Direction

Both the cost engineering and recycling of thermoset composite materials have received attention from the research community only as independent research areas. A comprehensive literature review concerning this area of research revealed that there is a lack of research efforts in developing the cost modelling of composites recycling and disposal. Predicting costs of recycle and disposal of the composite components, which encompass environmental and logistics costs, is a challenging research topic. The data related to the recycling and disposal of composites is far scarcer compared to that of metallic components.

The processes for recycling fiber-reinforced composites, such as pyrolysis, mechanical treatment, and solvolysis, vary in terms of effectiveness, costs, and environmental impacts. The appeal of these processes is subject to the costs they will incur during execution, hence, several works were published discussing the financial viability of the recycling techniques. Currently, most of the works concluded that proposed recycling methods for composites are still not financially attractive compared to traditional disposal routes, such as landfilling and incineration. However, with changing legislation towards the green economy and the potential increase of landfilling taxes, a profound shift towards recycling rather than landfilling will be inevitable in the long-term. Additionally, there is a scope for improving the fiber recovery rates and their structure in order to be able to use recovered products for high-value applications.

Further developments in the cost modelling of the recovery of fiber-reinforced composites can be accomplished through future research efforts. Cost modelling should take into consideration the impact of uncertainty and optimization on cost. The development of knowledge-based cost modelling systems will support design and manufacturing teams in understanding various cost elements. The financial viability of recycling composites is highly dependent on the selected recycling method and its parameters. All recycling techniques result in different costs; that is why it is important to adopt a suitable technique with optimized process parameters for recycling. The recovery rates of fibers and their market values should be aligned with the current industrial market. For instance, the assumption of 100% recovery for pyrolysis and solvolysis processes may diverge the actual results. It is essential to develop a standard LCC framework for recycling fiber-reinforced composites so that the study results of different institutions could be combined to gain an in-depth evaluation of recycling costs of carbon and glass-fiber composites. LCC can provide valuable insight for the selection of disposal route; however, the life cycle approach is usually more dedicated to the first three stages of the product life cycle, namely development, manufacturing, and use phase. A very limited number of studies were found examining the recycling phases. It is possible to analyze the disposal routes of composites from a cost-benefit analysis perspective. This is a more project-based approach focused on a particular time, whereas LCC spreads its efforts amongst all phases of the product. It is important to consider profit margins for recycling companies in the analysis, as the interest of all stakeholders in the recycling business should be taken into consideration. Equally, apart from cost breakdown structures for dismantling and recycling processes, salvage values of end-of-life products should be taken into consideration. Until today, cost modeling has been dependent on many assumptions about costs, such as the value of the recovered product, operating and maintenance costs, and equipment, etc. Therefore, to have accurate results from cost modelling, exogenous factors such as dismantling costs, packaging costs, and transportation costs should not be neglected. Further studies should include these factors that might affect the economic viability of the process. There is a need to integrate industry 4.0 areas such as digital twin and IoT with cost modelling of carbon fiber composites recycling.

## Figures and Tables

**Table 1:** Technical and economic barriers to composites recycling.

Technical	Poor recyclability due to the structure of the materials. In thermoset composites materials are cross-linked and cannot be remelted
	The great diversity of composite material types and mixtures hinders the development of a standardized waste collection and sorting procedure
	Composite materials contain cores and coatings separation of which requires a significant amount of manpower
Supply Chain	Transportation costs incurred by transporting the composite waste to recycling facilities
Market	A lack of commercially viable recycling methods. The energy intensity of some recycling methods pose a threat to financial viability
	The quality of recycled composites is viewed to be lower in contrast to virgin composites. This limits the application opportunities

**Table 2:** Recent studies on cost modelling for composites recycling.

Author	Publication Date	Process	Material Type	Main Findings
Hedlund-åström [72]	2005	Mechanical, pyrolysis, fluidized-bed, hydrolysis	Carbon fiber composites and other	The important factor of LCC is to choose which material will be replaced between virgin CF and glass fiber. Incineration is advantageous if the rCF is used instead of cheap materials such as glass fiber. The recycling technique parameters also have an impact on the quality of recovered fibers, which is one of the essential parameters in cost modelling
Li, Bai and Mckechnie [73]	2016	Mechanical treatment	Carbon fiber composite	The authors have performed LCC analysis of the mechanical recycling process for the recovery of carbon fiber composites. Additionally, they compared incineration and landfilling options for the UK. The study found that the mechanical recycling of CFs is not financially feasible with 4100 USD/ton compared to conventional ways of waste treatment. This is explained by the high costs of disassembly of parts and treatment processes. Also, the recovery rates are low with the market values of rCFs being relatively low
Meng et al. [66]	2018	Fluidized-bed process	Carbon fiber composite	CF can be recovered at 5 USD per kg which matches to 15% of the cost of virgin CF. In turn, rCFs show further cost reductions compared to virgin CF and conventional materials such as steel and aluminum
Vo Dong, Azzaro-pantel and Cadene [58]	2018	Grinding, pyrolysis, microwave and supercritical water	Carbon fiber composite	The model proposed compares landfilling, incineration, mechanical recycling, pyrolysis, and chemical recycling methods in terms of cost per weight of material. Grinding found to be viable for carbon fibers at moderate capacity (2000 t/year). Solvolysis was found to be the most expensive disposal route. Other recycling techniques were found to be highly dependent on the capacity and recovery rate of fibers due to high treatment costs
Hagnell and Åkermo [74]	2019	Pyrolysis, mechanical recycling, and co-processing in cement kilns	Carbon and glass fiber composites	The study developed a closed-loop model for lignin-based carbon fibers and glass fibers. Results showed that 50% of material cost reductions are possible with similar mechanical performance when using rCF instead of virgin CF in certain applications. Lignin-based CF are recommended to be incinerated to obtain energy, while glass fibers are ideal to reduce the cost of material by 50%
Shehab et al. [75]	2021	Mechanical, pyrolysis, fluidized-bed	Carbon fiber composite	The authors proposed fuzzy-logic based cost modelling for LCC of recycling CFRP material. The model also considers uncertainty factors and helps to choose the most cost-effective method
Shehab et al. [76]	2021	Mechanical recycling, pyrolysis, fluidized bed, and supercritical water	Carbon fiber composite	The paper presents a software tool that allows to calculate the CFRP recycling cost without any prior knowledge about recycling methods. The tool optimizes the choice of recycling method according to user inputs based on TOPSIS method

**Table 3:** Carbon fiber quality and cost of CFRP recycling methods.

Recycling Method	Tensile Strength Retention	Fiber Length (mm)	Processing Temperature (C)	UCF ($/kg)
Mechanical	N/A	0.001–10 [28]	N/A	0–3.5 [75]
Pyrolysis	4% to 85% of virgin [38]	Can be retained	350–1000 [28,38]	1.6–5.5 [75]
Fluidized bed	25% of virgin [38]	25 [24,35,38]	450–550 [35,38,79]	5 [79]
Solvolysis	>90% (near virgin) [38]	3–90 [28]	65–400 [28,38]	14–28 [75]
